# Characterization of *Brassica rapa* metallothionein and phytochelatin synthase genes potentially involved in heavy metal detoxification

**DOI:** 10.1371/journal.pone.0252899

**Published:** 2021-06-04

**Authors:** Jiayou Liu, Jie Zhang, Sun Ha Kim, Hyun-Sook Lee, Enrico Marinoia, Won-Yong Song

**Affiliations:** 1 International Research Center for Environmental Membrane Biology, Department of Horticulture, Foshan University, Foshan, Guangdong, China; 2 College of Agriculture and Life Sciences, Chungnam National University, Daejeon, Korea; Universidade de Coimbra, PORTUGAL

## Abstract

*Brassica rapa* is an important leafy vegetable that can potentially accumulate high concentrations of cadmium (Cd), posing a risk to human health. The aim of the present study was to identify cadmium detoxifying molecular mechanisms in *B*. *rapa* using a functional cloning strategy. A cDNA library constructed from roots of *B*. *rapa* plants treated with Cd was transformed into the Cd sensitive yeast mutant strain DTY167 that lacks the yeast cadmium factor (*YCF1*), and resistant yeast clones were selected on Cd containing media. Two hundred genes potentially conferring cadmium resistance were rescued from the surviving yeast clones and sequenced. Sequencing analysis revealed that genes encoding for metallothionein (MT)1, MT2a, MT2b and MT3, and phytochelatin synthase (PCS)1 and PCS2 accounted for 35.5%, 28.5%, 4%, 11.3%, 18.7% and 2%, respectively of the genes identified. *MTs* and *PCSs* expressing DTY167 cells showed resistance to Cd as well as to Zn. *PCS1* expressing yeast cells were also more resistant to Pb compared to those expressing *MTs* or *PCS2*. RT-PCR results showed that Cd treatment strongly induced the expression levels of *MT*s in the root and shoot. Furthermore, the different *MTs* and *PCSs* exhibited tissue specific expression. The results indicate that *MTs* and *PCS* genes potentially play a central role in detoxifying Cd and other toxic metals in *B*. *rapa*.

## Introduction

Cadmium (Cd), a human carcinogen (Group 1), is widely distributed in the earth’s crust (0.1 mg/kg) [1]. For humans, the main routes of exposure to Cd are smoking of cigarettes and ingestion of food [2, 3]. Grains and leafy vegetable account for 56–59% and 9–10% of dietary Cd intake, respectively, for Chinese population [4]. Ingestion of food contaminated with Cd can strongly increase cadmium concentration in human bodies [5]. Cd induces oxidative damage of DNA, which is caused by over-production of ROS, leading to reduction of cellular proliferation, inhibition of apoptotic mechanisms and blocking of the DNA repair mechanisms [6, 7]. These damages can cause the development of cancer and damage of several organ systems such as the kidney (proteinuria, kidney stones, glomerular and tubular damage), the respiratory system (Pneumonitis), the reproductive system (testicular necrosis), and the skeletal system (Itai-Itai disease) [8]. In plants, exposure to Cd can also lead to deleterious effects in growth and development. It induces growth retardation caused by inhibition of photosynthetic activity, stomatal movement, enzymatic activities, and nutrient transportation [9]. Therefore, plants have developed several defense mechanisms to cope with the presence of Cd, such as regulation of Cd transporters involved in Cd uptake, efflux or allocation, and detoxification of cellular Cd through the sequestration or chelation, which decreases the concentration of free Cd, and hence decreases toxic reactions. However, different plants show different sensitivities against a given Cd concentration.

The plasma membrane of roots plays a crucial role in reducing Cd uptake or activating Cd extrusion from the root to the soil. Depending on the plant species a variable proportion of Cd taken up by roots is translocated to the shoots by divalent cation transporters located at the plasma membrane of the stele in root cells. Cd is mainly taken up by root-localized transporters of the ZIP and NRAMP family. AtIRT1, the first ZIP transporter characterized in *A*. *thaliana* [10], is exclusively expressed at the root epidermis under iron deficiency conditions [11], and besides iron transports Cd(II) as well as Zn(II) [12]. Arabidopsis overexpressing *AtIRT1* is hypersensitive to Cd due to the increased accumulation of Cd when compared to the WT [13]. Rice Nramp5 takes up Cd(II)/ Mn(II) at the distal side of both exodermis and endodermis of roots. In Nramp5 knockout plants Cd uptake is strongly decreased, and Cd concentration in shoots and grains are lower in these plants [14]. Arabidopsis Plant cadmium resistance protein (AtPCR) a PLAC8 motif-containing protein family located at the plasma membrane. Overexpression of *AtPCR1* and *AtPCR2* highly enhances Cd tolerance and inhibits Cd accumulation in yeast and Arabidopsis, while *AtPCR2* knockout plants are sensitive when exposed to an excess of Cd, Zn, Cu and Fe, due to a reduction of the metal export activity into the rhizosphere [15, 16].

When Cd enters a plant cell, chelation is the primary and crucial response to reduce its toxicity. The most important chelation reaction for Cd is the binding to thiol groups. Phytochelatins (PCs) and metallothioneins (MTs) are major Cd metal-binding ligands and have been characterized well as Cd resistance factors of plants. PCs synthesis is rapidly activated by Cd, Cu, Zn, Ag, Au, Hg, and Pb [17, 18]. Genes for the synthesis of PCs have been identified in Arabidopsis and wheat [19, 20]. The phytochelatin synthase (PCS*)* deficient Arabidopsis mutant *cad1* is hypersensitive to Cd, suggesting that PCs are crucial molecules to detoxify Cd in Arabidopsis. Interestingly, PCS activities are regulated both at the transcriptional and post transcriptional level. *PCS1* mRNA levels of Arabidopsis are not influenced by Cd exposure [19], while expression of *PCS1* of wheat is increased by Cd treatment [20]. In contrast to PCs, MTs, low molecular weight cysteine-rich proteins, are produced by mRNA translation. MTs are classified into 4 types according to their structures, MT1, MT2, MT3, and MT4 [21, 22]. To understand the heavy resistance mechanisms in plants, MT genes have been intensively studied in Arabidopsis and other plants. AtMT1, AtMT2a and AtMT3 induce Cd(II) tolerance in yeast and *Vicia faba* guard cells transformed with these genes [23, 24]. Expression of *Brassica juncea MT2* (*BjMT2*), *Cajanus cajan MT1* (*CcMT1*) and *B*. *campestris MT1* and *MT2* (*BcMT1* and *BcMT2*) enhances Cd and Cu tolerance in *A*. *thaliana* [25–27]. Arabidopsis MT4a and MT4b regulate homeostasis of Cu and Zn in Arabidopsis seeds [28].

*Brassica* species are important crops supporting valuable nutrients for humans, such as oil, vitamins, glucosinolates, soluble sugars and carotenoids [29]. Moreover, they are known as heavy metal tolerant and accumulating plants [30]. *B*. *juncea* (indian mustard) is a potential plant to clean-up soil contaminated with heavy metals, because it is a high-biomass-producing crop with the character of a heavy metal accumulator [30]. *B*. *napus* accumulates more Cd and Zn in shoots than roots, indicating effective translocation of Cd and Zn into shoots [30–32]. Flowering Chinese cabbage (*B*. *rapa*) efficiently accumulates more Cd than Hg and Cr [33]. However, it is not known which molecular mechanisms are involved in the different *Brassica* species in increasing heavy metal tolerance or accumulation.

*B*. *rapa*, a diploid *Brassica* species carrying an AA genome, is considered as one of the most important crop models due to the high level of genetic resemblance with Arabidopsis and containing the smallest genome size among the *Brassica* species [34, 35]. *B*. *rapa* is a major leafy vegetable crop for Asian, especially Korean eating kimchi made from this vegetable. However, since *B*. *rapa* potentially accumulates high concentrations of heavy metals it may cause serious health problems in people eating this crop. In order to develop *B*. *rapa* accumulating lower level of toxic metals, it is therefore important to elucidate how this plant takes up heavy metals and which heavy metals are preferentially accumulated. Here we screened for *B*. *rapa* genes participating in Cd tolerance using a yeast expression system, and revealed the molecular function of the corresponding genes. This work provides information about which major molecules maintain Cd tolerance when *B*. *rapa* is exposed to Cd.

## Materials and methods

### Plant culture and cDNA library construction

*B*. *rapa* cultivar Maeruk seeds were sown on soil or sand with a diameter of 2 mm supplemented with 1/5 strength Hogland liquid medium and grown in a growth chamber under controlled conditions (16/8 light/ dark cycle at 26 °C). Three-week old hydroponic cultured *B*. *rapa* plants were treated with 50 μM CdCl_2_ for 2-24h and harvested to extract RNA. To develop flowers in *B*. *rapa*, two-week old soil grown plants were incubated in a low temperature incubator (16/8 light/ dark cycle at 26 °C) for 3 weeks, and then cultured under a 16 h/8 h light/ dark cycle at 26 °C until flowering.

Total RNA was extract using the phenol/chloroform method [15]. mRNA was isolated from total RNA using an mRNA isolation kit (Promega). To construct the cDNA library, cDNA was synthesized from mRNA and fractionated according to the protocol of ZAP Express cDNA Synthesis Kit (Agilent). Fractionated cDNA (0.7–5 kb) was inserted into the EcoRI/XhoI sites of yeast shuttle vector pYES2 (Thermo Fisher), and then transformed into *E*. *coli*. The average insert size of the library was estimated using PCR with specific primers of the pYES2 vector.

### Screening for Cd tolerance genes from *B*. *rapa* cDNA library

To isolate genes implicated in Cd tolerance from the *B*. *rapa*, the cDNA library was transformed into the Cd sensitive yeast mutant DTY167 (MATα *ura3-52 his6 leu2-3*,*112 his3-Δ*,*200 trp1-901 lys2-80 suc2^-^ Δycf1∷hisG*). The transformants were cultured on synthetic dextrose without uracil (SD ura-) agar plates at 30 °C for 3 days, harvested all together, and then spread on synthetic galactose agar plates without uracil (SG ura-) supplemented with 70 μM CdCl_2_. After 5 days of culture in a 30 °C incubator, surviving yeast colonies were recovered on a SD ura- agar plate for 1 day, streaked on both SD ura- and SG ura- agar plates containing 40 μM CdCl_2_ to discard false positive colonies. An insert in the pYES2 vector is expressed by galactose, and suppressed by dextrose, therefore yeast colonies showing Cd tolerance on the SD ura- medium are false positive. Total DNA was extracted from Cd tolerance yeast colonies, *B*. *rapa* cDNA was amplified using PCR with the total DNA as template and the specific primer set of pYES2 vector, and then PCR products were sequenced.

### Analysis of Cd tolerance genes sequences

Coding sequence (CDS) and amino acid sequences from DNA sequences of Cd tolerance genes were determined using a nucleotide sequence translation program (https://web.expasy.org/translate/). CDS and amino acid sequences of Cd tolerance genes were applied to blast and blastp (National Center for Biotechnology Information; https://blast.ncbi.nlm.nih.gov/Blast.cgi) to identify the orthologues in *Brassica* species and Arabidopsis. CDS and amino acid sequences from MTs and PCSs genes of *A*. *thaliana* and various *Brassica* species were subjected to multiple protein sequence alignment using the ClustalW software (BioEdit or Mega-X). To develop Neighbor-joining phylogenetic trees, alignments of DNA and amino acid were subjected to MEGA X program [36]. The bootstrap values (percentage) of 1000 replicates are shown at the branching points. Zn binding amino acid residues of BrMTs were identified using the ZincBinder program (http://proteininformatics.org/mkumar/znbinder/).

### Heavy metal tolerance test

Plasmids harboring Cd resistance genes were rescued from Cd resistance yeast clones and retransformed into the DTY 167 yeast strain using the LiAc/PEG method. The transformed yeast strains were cultured in SD ura- liquid medium for 16h, pelleted using centrifugation, washed using autoclaved distilled water (ADW), and then diluted according to the optical cell density (OD600). The serial diluted yeast cells were spotted on SG ura- agar plant supplemented with 40 μM CdCl_2_, 5 mM ZnCl_2_ and 1.2 mM Pb-tartarate, and grown in a 30 °C incubator.

### Expression analysis of Cd tolerance genes in *B*. *rapa*.

To analyze the tissue specific and Cd- inducible expression of Cd tolerance genes, qRT-PCR was performed. Flowers, stems and leaves were harvested at the reproductive stage of plants grown on soil, and roots and rosette leaves were collected from one-month old plant cultured in a hydroponic system with 1/5 MS medium. To analyze the Cd inducible expression, one-month old *B*. *rapa* plants were treated with 100 μM CdCl_2_ for different times and harvested. Total RNA was extracted using Takara MiniBEST Plant RNA extraction Kit (Takara Co.), and cDNAs were synthesized from RNA using the RevertAid First Strand cDNA Synthesis Kit (Thermo Fisher). The qRT-PCR was performed using Light Cycler 48II (Roche Life Science) with gene-specific primer sets (S1 Table), cDNA, and TG Green Premix Ex Taq II (Takara Co.). PCR conditions were 55 cycles of 95°C for 10 sec, 53°C for 20 sec, and 72°C for 20 sec. The expression level of each gene was normalized by that of actin.

### Accession numbers

Sequence data from this article can be found in the GenBank/EMBL databases under the following accession numbers: MT361642 for BrPCS1_1, MT361643 for BrPCS2_1, MT361644 for BrMT1a, MT361645 for BrMT1b, MT361646 for BrMT1c, MT361647 for BrMT2a, MT361648 for BrMT2b, MT361649 for BrMT3, S71334.1 for BnMT1, AF458412.1 for BoMT1, NM_100634.2 for AtMT-1c, AF386921.1 for AtMT1a, NM_001037008.3 for AtMT1b, AY486004.1for TcMT1, NM_111773.4 for AtMT2A, AK227568.1 for AtMT2b, Y10850.1 for BjMT2a, AF200712.1 for BoMT2a, XM_013767061.1 for BoMT2-2b, Y10851.1 for BjMT2b, AY486002.1forTcMT2b, NM_112401.2 for AtMT3, AB057413.1 for BjMT3a, AB057414.1 for BjMT3b, AB057415.1 for BjMT3c, XM_013847349.1 for BnMT3a, XM_013770994.1 for BoMT3c, XM_013842239.2 for BnMT3b, XM_013825697.2 for BnMT3c, XM_013782575.1 for BoMT3b, XM_009103491.2 for BrPCS1, XM_009120432.2 for BrPCS2, XM_013742676.1 for BnPCS1, XM_013742676.1 for BoPCS1, BAB85602.1 for BjPCS1, NP_199220.1 for AtPCS1, XP_013660298.1 for BnPCS2, XP_013602398.1 for BoPCS2, and NP_171894.1 for AtPCS2.

## Results

### Screening of Cd tolerance genes in *B*. *rapa*

**Fig 1:**
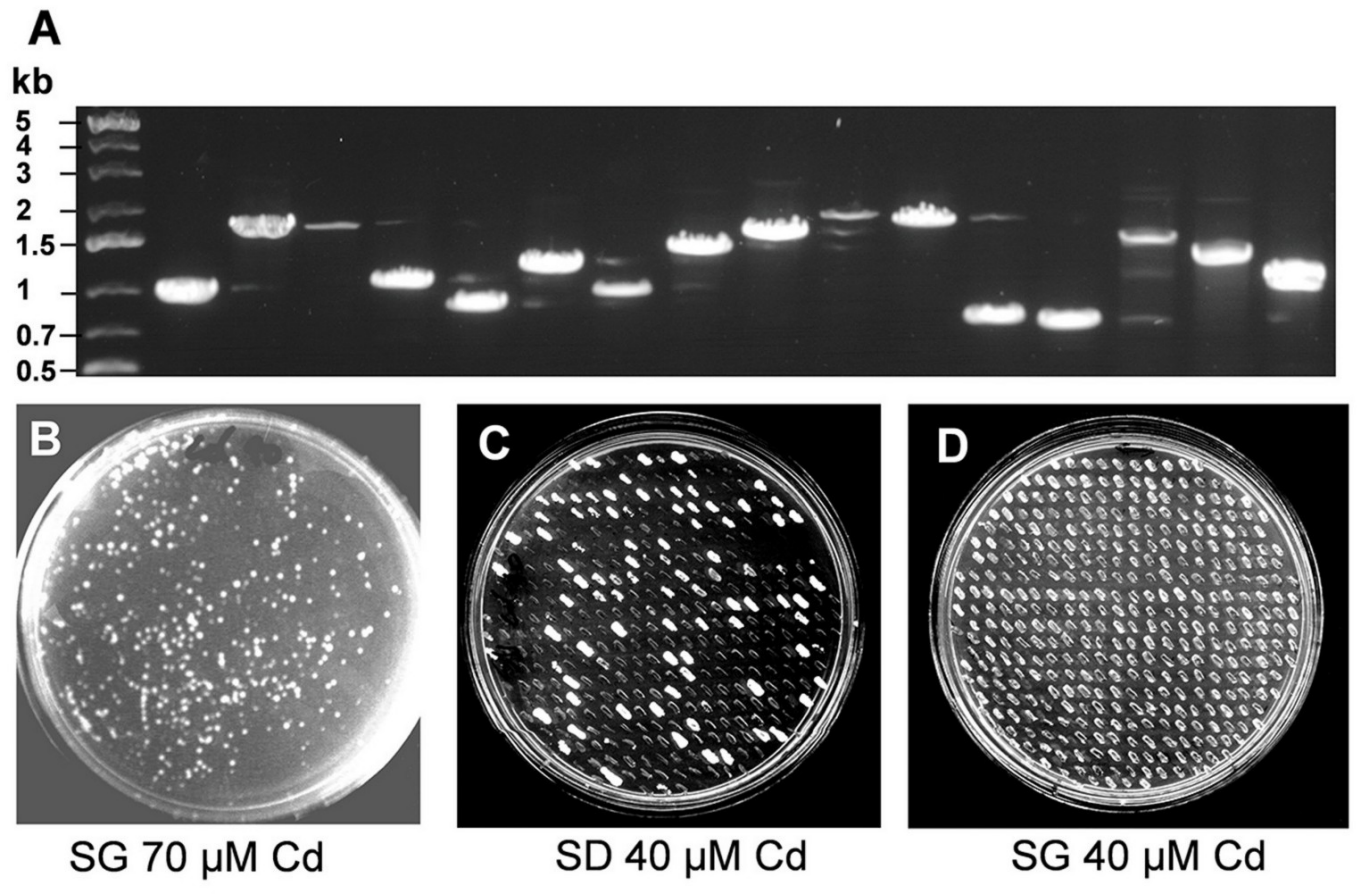
Screening of Cd tolerance gene in *Brassica rapa*. (A) Insert size of *B*. *rapa* cDNA library. Inserts of the *B*, *rapa* cDNA library in the pYES2 vector were amplified using PCR with vector linker primers, and electrophoresed in 1% agarose gel. (B) First selection of Cd tolerance genes in *B*. *rapa*. The Cd-sensitive yeast mutant (DTY167) was transformed with a cDNA library of *B*. *rapa* and grown on synthetic galactose (SG) agar plates supplemented with 70 μM CdCl_2_ to select clones harboring Cd tolerance genes. (C, D) Second selection of Cd tolerance genes in *B*. *rapa*. The surviving clones from the 1’st screening were grown on synthetic dextrose (SD) and synthetic galactose (SG) media supplemented with 40 μM CdCl_2_ to select real positive clones. False-positive clones surviving on SD supplemented with CdCl_2_ were discarded. The clones growing on SG but not on SD were further analyzed.

We developed a yeast expression system to reveal genes participating in Cd tolerance in *B*. *rapa*. Briefly, we synthesized a cDNA using mRNA extracted from roots of *B*. *rapa* plants treated with Cd, and constructed a cDNA library using the yeast expression vector pYES2. The average size of inserts in the vector was approximately 1.5 kb (Fig 1A, S1 Fig). To identify genes conferring Cd tolerance, we introduced the library into the Cd sensitive *ycf1* yeast strain DTY167 and picked the yeast colonies that grew on synthetic galactose (SG) medium containing 70 μM Cd(II) (Fig 1B). After the first screening procedure, clones showing Cd tolerance in both synthetic dextrose (SD) medium and SG medium were notified as false-positive and discarded since the inserted genes should not be expressed in the presence of dextrose, because the genes are under the control of the GAL4 promoter (Fig 1C). However, it should be mentioned that since we used full length cDNA clones that include the UTR regions, it is unlikely, but cannot be excluded that some UTRs contain regulatory elements that would allow for expression of the gene in the absence of Gal and hence that we missed some novel Cd tolerance gene using this approach. Finally, we randomly selected 200 colonies, and sequenced the inserts to identify *B*. *rapa* genes participating in Cd tolerance in this yeast mutant. Among the Cd tolerance genes identified, metallothionein (*MT*)1, *MT2a*, *MT2b*, *MT3* accounted for 35.5%, 28.5%, 4% and 11.3% respectively, and phytochelatin synthase *(PCS*)1 and 2 accounted for 18.7% and 2% (Fig 1). However, we could not find *BrMT4* genes in this screen, very probably due to the low expression of *MT4* in vegetative tissues including roots [28, 37].

Here, we identified 3 different *BrMT1* genes, *BrMT1a*, *BrMT1b*, and *BrMT1c*, and their CDSs shared a high homology to each other, but their 3’ UTRs were quite variable (~ 30% identity) (S2 Fig). CDSs of *BrMT2a* and *BrMT2b* exhibited 81% (201/249) DNA sequence identity, but their 5’ UTRs were more distinct to each other (60% identity (37 nt/61 nt)) (S2 Fig). At the amino acid level, BrMT2a and BrMT2b exhibited an identity of 79% (65aa/82aa) (S3 Fig). Besides the metallothioneins we identified two different phytochelatin synthase (*BrPCS*) genes present at high frequency in the pool of the 200 genes sequenced (Fig 1B). *BrPCS1_1* (MT361642) exhibited 99% (1742/1765nt) identity with *B*. *rapa* glutathione gamma-glutamylcysteinyltransferase 1 (*BrPCS1*; XM_009103491.2), and *BrPCS2_1* (MT361643) had 99% (1640/1658nt) with *B*. *rapa* glutathione gamma-glutamylcysteinyltransferase 2 (*BrPCS2*; XM_009120432.2) (data not shown). The BrPCS1_1 protein exhibited also 99%-98% identity with *B*. *napus* PCS1 (XM_013850984.2), *B*. *oleacea* PCS1 (ADD37638.1) and *B*. *juncea* PCS1 (BAB85602.1), while it showed 92% identity with the Arabidopsis PCS1 (NP_199220.1). BrPCS2_1 exhibited 99% - 96% identity with PCS2s of *B*. *napus* (XP_013660298.1) and *B*. *oleracea* (XP_013602398.1)), whereas it had 89% and 75% identity with *A*. *thaliana* PCS2 (NP_171894.1) and *B*. *rapa* PCS1 (RID60624.1) (S4 Fig).

**Fig 2:**
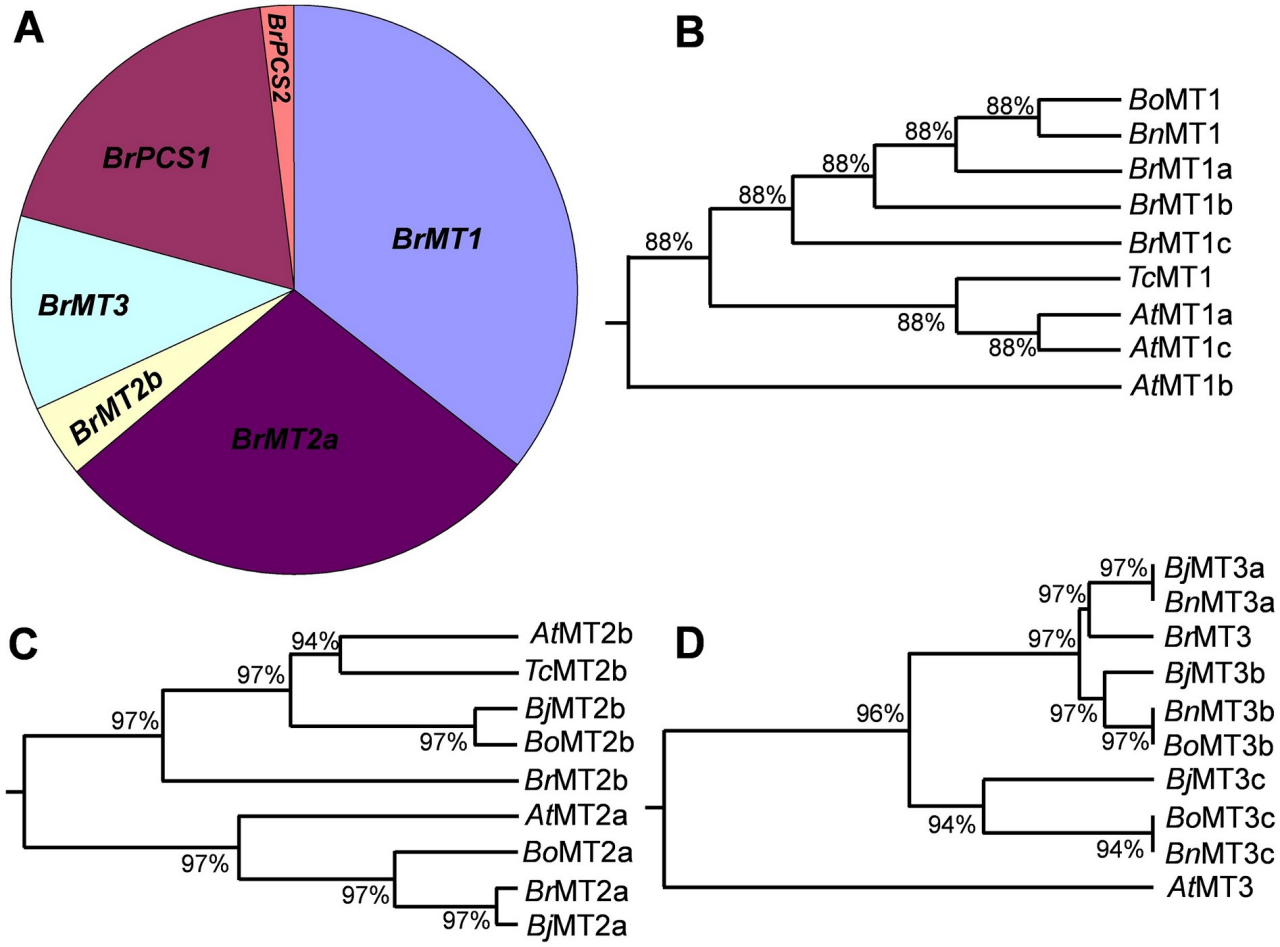
Relative frequency of Cd tolerance genes and phylogenetic trees of BrMTs in Brassica family. (A) Relative frequency of Cd tolerance genes in *B*. *rapa*. Cd tolerance genes were identified from 200 yeast clones exhibiting Cd tolerance, and the relative frequency of Cd tolerance genes was analyzed. *(B-D)* Phylogenetic analysis using the neighbor-joining method implemented in MEGA X. The results are based on multiple alignments of MTs amino acid sequences. The bootstrap values (percentage) of 1000 replicates are shown at the branching points.

To investigate the evolutionary relationships of BrMTs with MTs from *A*. *thaliana*, *Brassica* species and *Thlaspi caerulescens*, we constructed a phylogenetic tree based on the alignment of cDNA sequences of CDS and amino acid sequences of MTs. The phylogenetic trees based on amino acid and CDS sequences exhibited the same pattern (Fig 2B–2D, S5 Fig). BrMT1s are grouped with the *Brassica* family, and are separated from AtMT1s and TcMT1 (Fig 2B). However, BrMT2a and BrMT2b are not grouped together, and they group with subgroups from the *Brassica* family, *A*. *thaliana* and *T*. *caerulescens* (Fig 2C). BrMT3 groups with MT3a subfamily members of the *Brassica* family and *A*. *thaliana* (Fig 2D), indicating that BrMT3 is belonging to the a-subgroup of MT3.

### Tissue specific expression of Cd tolerance genes

**Fig 3:**
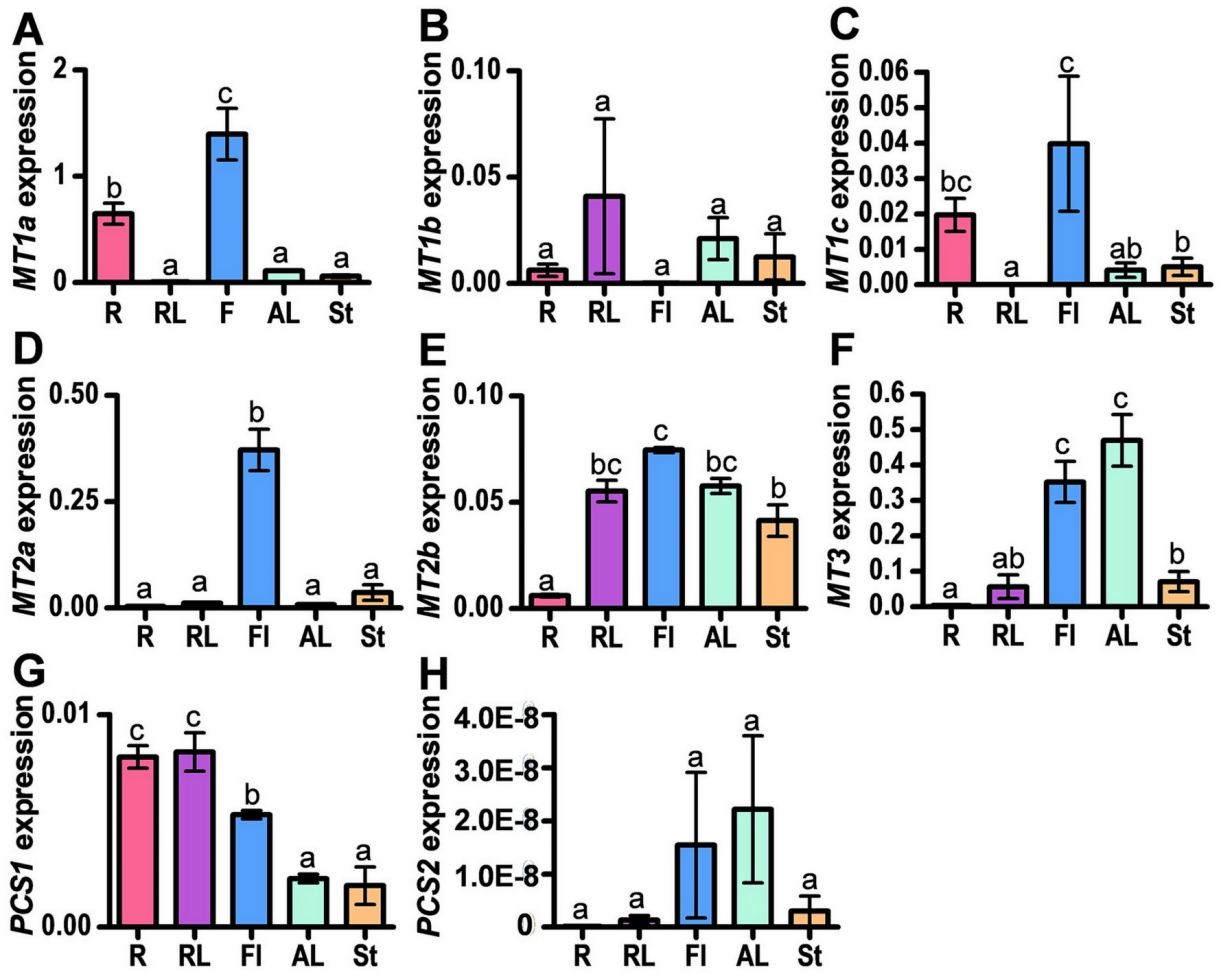
Tissue specific expression of *BrMTs and BrPCSs* in *B*. *rapa*. To analyze the expression of *B*. *rapa* Cd tolerance genes (*BrMTs* and *BrPCSs*), qRT-PCR was performed with cDNA synthesized from total RNA as a template and gene-specific primers (S1 Table). The expression levels of Cd tolerance genes were normalized to the level of actin, and the values indicate the average and standard error (biological replicates: 3). Different letters indicate that the means (between various samples) are significantly different by the Tukey HSD test (P≤0.05). R, roots from one-month old plants; RL, rosette leaves from one-month old plants; Fl, flower; AL, leaf from flowering plants; St, stem from flowering plants.

MTs and PCs, are crucial elements in regulating the homeostasis of essential and nonessential metal ions in plants. MT genes of the *Brassica* family are specifically expressed in various tissues. *B*. *campestris MT1* (*BcMT1*) was expressed predominantly in roots, whereas *BcMT2* was expressed mainly in leaves [25]. To understand the potential biological roles of the genes putatively implicated in Cd tolerance from *B*. *rapa* identified in this work, we examined their expression patterns in leaves (rosette leaves) and roots at the vegetative stage, and leaves, stems and flowers at the reproductive stage of *B*. *rapa*. Each *MT1* gene had a distinct expression pattern (Fig 3A–3C); *BrMT1a* was highly expressed in roots and flowers, *BrMT1b* was expressed in all tissues tested in this study, and *BrMT1c* was mainly expressed in roots and flowers. *BrMT2a* was highly expressed in flowers, and *BrMT2b* was expressed in all tissues examined in this study, especially at a higher level in rosette leaves, flowers, cauline leaves and stems (Fig 3D and 3E). Expression of *BrMT3* was mainly observed in flowers and leaves (Fig 3F). *BrPCS1* was expressed in all tissues (Fig 3G), similarly as reported for Arabidopsis *PCS1* [19]. We found that expression levels of *BrPCS2* were lower than those of *BrPCS1* (Fig 3H). Tissue specific expression of *BrMTs* and *BrPCSs* suggest that each *BrMTs* and *BrPCSs* exhibits specific tasks in heavy metal tolerance or homeostasis in *B*. *rapa*.

### Cd-dependent expression of Cd tolerance genes

**Fig 4:**
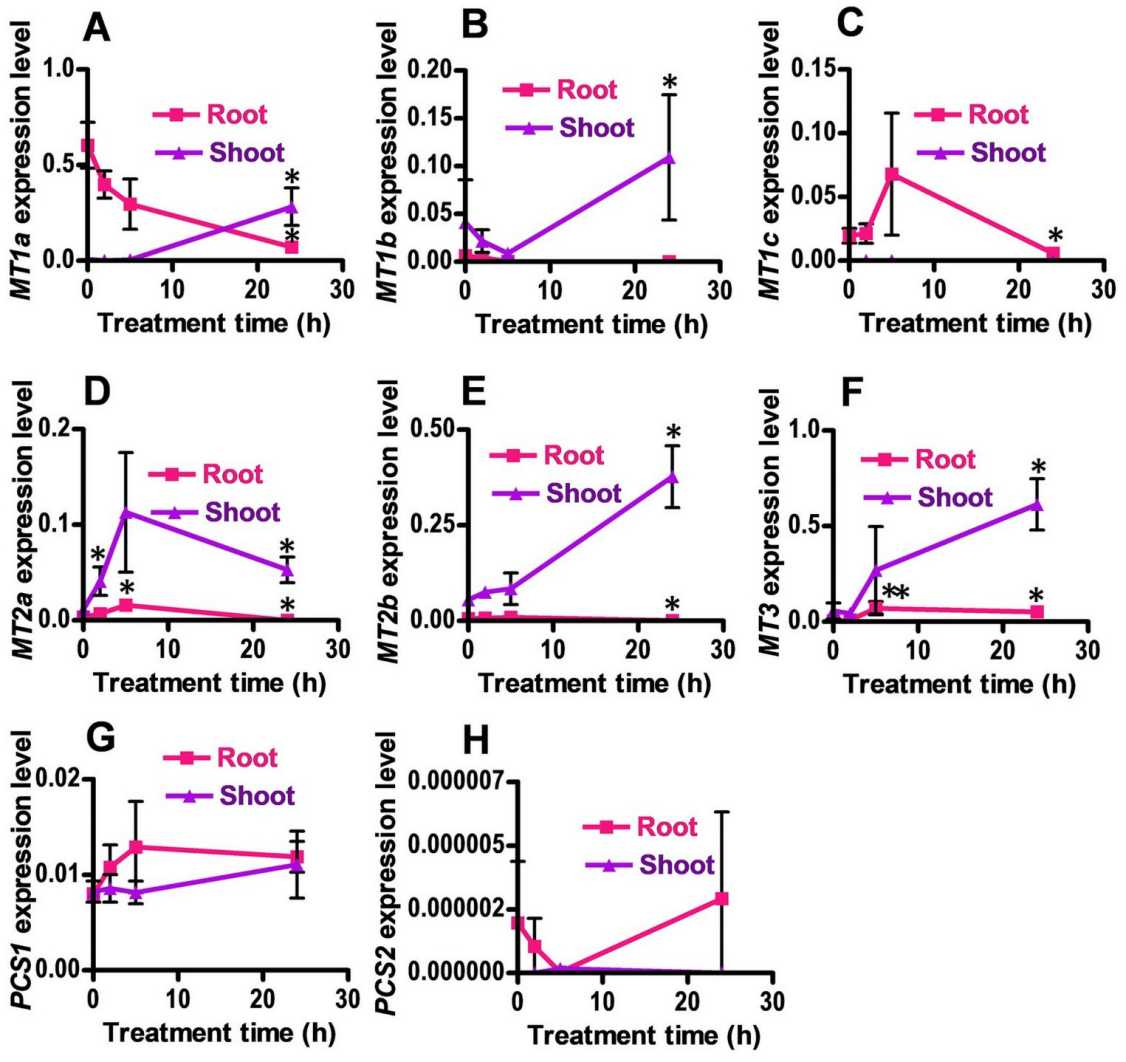
Cd-inducible expression of *BrMTs and BrPCSs* in *B*. *rapa*. One-month old *B*. *rapa* plants were treated with 100 μM CdCl_2_ for different times and harvested to analyze the expression levels of *B*. *rapa* Cd tolerance genes. qRT-PCRs were performed with cDNA synthesized from the RNA as a template and gene-specific primers (S1 Table). The expression levels of Cd tolerance genes were normalized to the level of actin, and the values indicate the average and standard error (biological replicates: 3). *P < 0.05, **P < 0.01 (Student’s t-test).

Over-expression of *MT* genes enhances Cd and Cu tolerance in plants. *Brassica BcMT1*, *BcMT2*, *BjMT2*, and pigeon pea *MT1*(*CcMT1*) enhance tolerance to cadmium and copper when they are overexpressed in *A*. *thaliana* [25–27]. AtMT2a and AtMT3 protected guard cell chloroplasts from degradation upon exposure to Cd(II) when they are overexpressed in *V*. *faba* guard cells by particle bombardment [24]. These results indicate that upregulation of *MT*s is important to increase heavy metal tolerance in plants. To know whether in *B*. *rapa MT*s and *PCS*s genes are upregulated to reduce the toxicity caused by an excess of heavy metals, we examined their transcript levels in response to Cd using qRT-PCR (Fig 4). *BrMT1a* exhibited the highest expression level among the three *BrMT1s*, and its expression was increased in shoots but decreased in roots in the presence of Cd (Fig 4A). Expression levels of MT1b, *BrMT2a*, *MT2b and BrMT3* genes were also increased in shoots in the presence of Cd (Fig 4B, 4D, 4E and 4F). In roots, the expression levels of MT1c and MT2a were increased when plants were treated with Cd for 5 h, but the expression levels declined with time of exposure (Fig 4C and 4D). Expression of *BrMT3* was induced in roots by Cd and persisted for 24h (Fig 4F). In roots, the expression pattern of *BrPCS1_1* appeared to be increased by Cd treatment, but the expression difference was not significant (Fig 4G). *BrPCS2_1* expression levels were very low, so we could not clearly determine whether *BrPCS2_1* was induced by Cd treatment (Fig 4H). The qRT-PCR results suggest that *B*. *rapa* might enhance Cd tolerance through the induction of Cd tolerance genes, such as *BrMTs* and *BrPCSs*.

### Enhanced heavy metal tolerance in yeast expressing Cd tolerance genes

**Fig 5:**
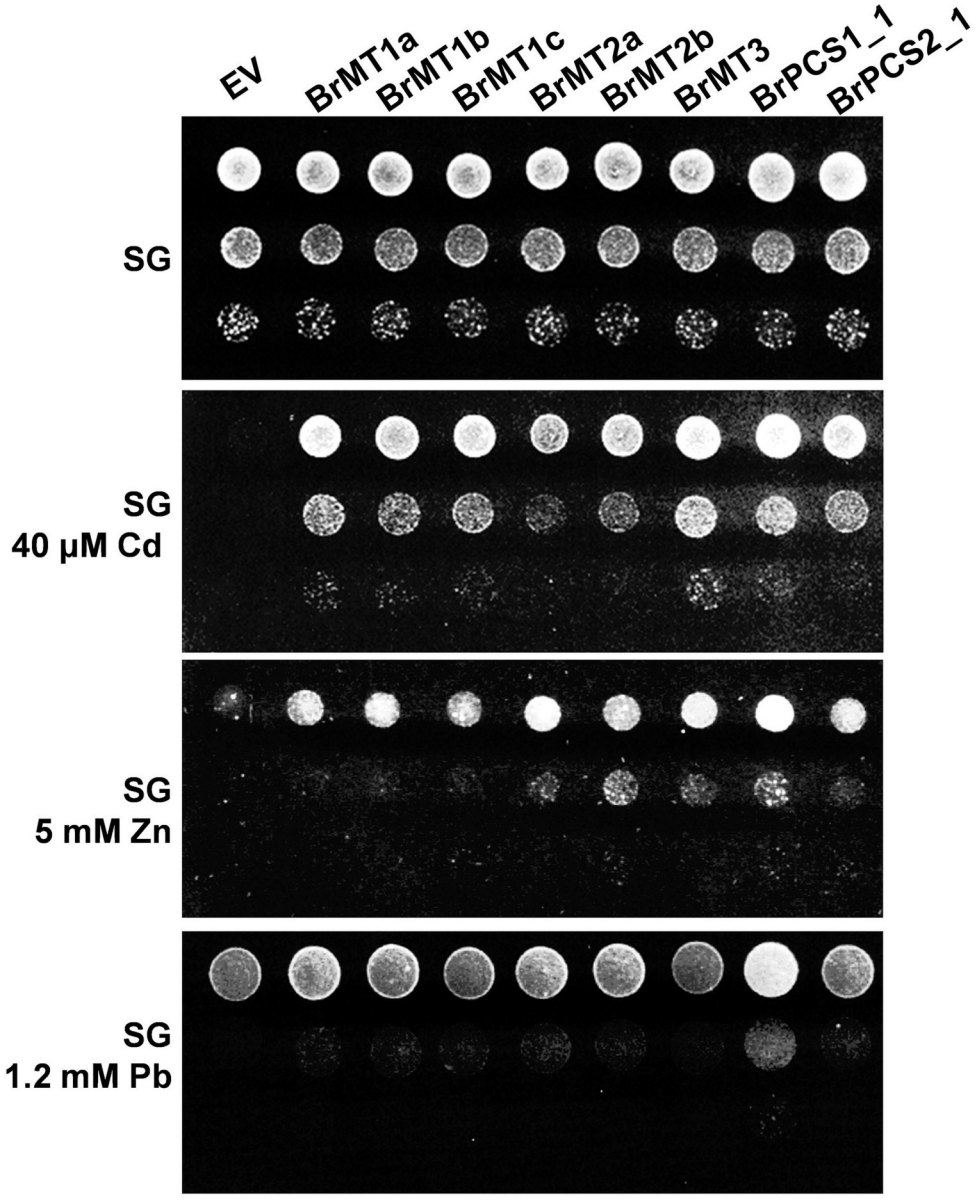
Enhanced heavy metal tolerance in yeast strains expressing *B*. *rapa* Cd tolerance genes. DTY167 yeast cells were transformed with empty vector (V), *BrMT1a*, *BrMT1b*, *BrMT1c*, *BrMT2a*, *BrMT2b*, *BrMT3*, *BrPCS1* and *BrPCS2*. The yeast strains were cultured in synthetic dextrose without uracil (SD ura−) liquid medium, spotted on synthetic galactose without uracil (SG ura−) agar plates supplemented with 40 μM CdCl_2_, 5 mM ZnSO_4_ or 1.2 mM Pb-tartrate, and cultured at 30 °C for 3–4 d.

In order to compare and examine the degree of tolerance which could potentially be conferred by the genes identified in this research, we expressed these genes in yeast and performed a phenotypic analysis on plates containing Cd, Zn and Pb (Fig 5). All yeast strains harboring *BrMT*s, *BrPCS*s and the empty vector exhibited comparable growth on synthetic galactose (SG) plates, but all yeast strains expressing *BrMT*s and *BrPCS*s showed dynamic growth patterns when exposed to an excess of heavy metals. All yeast strains expressing *BrMT*s and *BrPCS*s were more tolerant to Cd and Zn than the yeast strain harboring the empty vector. Yeasts expressing *BrMT3* and *BrPCS1* exhibited the highest Cd tolerance, whereas yeast strains expressing *BrMT2s*, *BrMT3* or *BrPCS1* performed the best under Zn excess conditions. In contrast, in the presence of Pb, a positive effect on growth could only be observed in yeasts expressing *BrPCS1*, but not in those expressing *BrMTs* and *BrPCS2*. This result suggests that Pb can induce PC synthesis only in yeast cells expressing BrPCS1, and that the two cysteine residues of C-terminus of BrPCS1, which are absent in BrPCS2, might be important to recognize Pb and synthesize PCs in response to the presence of Pb (S4 Fig).

**Fig 6:**
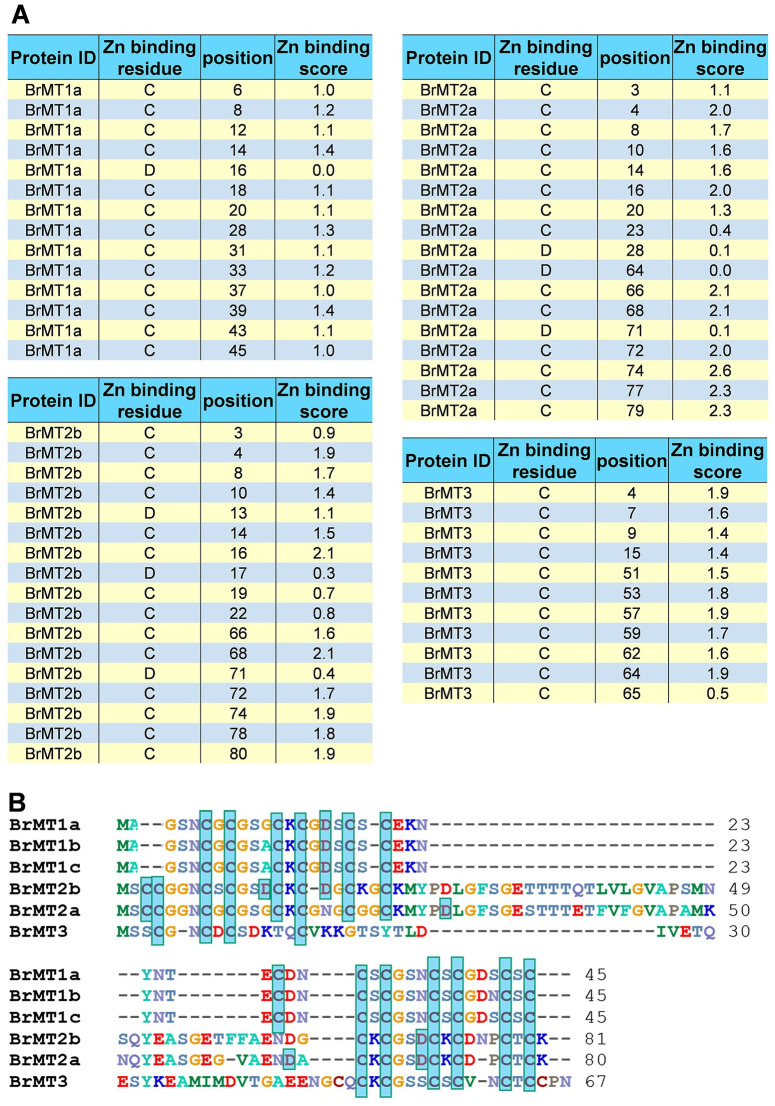
Analysis of Zn binding amino acid residues in *B*. *rapa* MTs. Zn binding amino acid residues of BrMT1a, BrMT2a, BrMT2b and BrMT3 were identified through the ZincBinder (http://proteininformatics.org/mkumar/znbinder/), and alignment of BrMTs was performed using CLUSTALW program of BioEdit. (A) Zn binding amino acid positions and Zn binding scores of BrMT1a, BrMT2a, BrMT2b and BrMT3. (B) Alignment of BrMT1a, BrMT2a, BrMT2b and BrMT3 protein. Boxes indicate putative Zn binding amino acid residues.

BrMT2s and BrMT3 were more efficient than BrMT1 in conferring Zn tolerance in yeast. This result suggests that BrMT2 and BrMT3 protein may contain a domain allowing this protein to increase Zn tolerance. To prove this question, we identified and compared Zn binding amino acid residues of BrMTs through ZincBinder (http://proteininformatics.org/mkumar/znbinder/). All BrMT1s, BrMT2s and BrMT3 proteins had 2 distinct putative Zn binding domains (Fig 6). BrMT1s have 14 putative Zn binding residues, BrMT2a and BrMT2b contain 17 amino acids capable to bind Zn(II), and BrMT3 has 11 amino acids that may act as putative Zn binding sites. BrMT2s and BrMT3 had higher Zn binding scores than BrMT1, indicating a stronger Zn binding force for BrMT2s and BrMT3 (Fig 6A). Interestingly, the Zn binding scores matched with the degree of Zn tolerance shown in yeast strains expressing *BrMT1*s, *BrMT2*s and *BrMT3* (Fig 5).

## Discussion

### BrMTs and BrPCSs are potentially major genes implicated in Cd detoxification in *B*. *rapa*

Genomes of *Brassica* species are closely related to that of the model plant *A*. *thaliana* [34, 35]. *B*. *juncea* (AABB genome) and *B*. *napus* (AACC genome) are well-known heavy metal accumulators, exhibiting a heavy metal tolerance phenotype. They share the *B*. *rapa* genome, because they are interspecific hybrids of *B*. *rapa* (AA genome) and *B*. *nigra* (BB genome) or *B*. *rapa* and *B*.*oleracea* (CC genome), suggesting that the heavy metal related characters of *B*. *juncea* and *B*. *napus* might be due to their common *B*. *rapa* genome. *B*. *rapa* carries the smallest genome size among the *Brassica* family and is considered as a model crop [34, 35]. For this reason, we attempted to identify the gene pool potentially implicated in conferring Cd tolerance in *B*. *rapa* by expressing a *B*. *rapa* cDNA library in yeast. Most studies published so far are focused on individual target genes [25–27]. The aim of our work was to get a general overview of the genes implicated in heavy metal tolerance and to provide a study that could link the different studies were the effect of individual genes are described. By sequencing 200 genes conferring Cd tolerance, we identified only heavy metal chelators, MTs and PCSs (Fig 2A), but not any metal ion transporters, such as MTPs, ABCs or a PCR [15, 38]. The results might be due to the abundance of MTs and PCS mRNA when plants are treated with an excess of Cd condition rather than a low quality of the cDNA library, since the average size of the inserts was approximately 2 kB (Fig 1a), and transcripts of *BrMTs* genes were highly induced in roots treated with Cd (Fig 4). In rice serial analysis of gene expression (SAGE), transcripts of *MT* genes accounted for almost 3% of the total transcripts in two-week-old seedlings [39].

*BrMT*s and *BrPCS*s increased strongly Cd and Zn tolerance in yeast, while Pb tolerance was provided only by *BrPCS1* (Fig 5). In Arabidopsis, PC synthesis is activated by Zn(II) and Pb(II) treatment, and *PCS1* nonsense mutants cad1s are hypersensitive to Zn(II) as well as Pb(II) [40, 41]. These results indicate that PCs are fundamental molecules to prevent cytosolic toxicity caused by Zn and Pb as well as Cd. While the role of PCs in conferring heavy metal tolerance is clearly established, the role of MTs in this role is still a matter of debate. Our results showed that BrMTs accounted for 79% of total genes identified in functional screening using yeast expression system with *B*. *rapa* cDNA library developed using plants treated with Cd(II) (Fig 1), together with the our results showing that several MTs are induced by Cd and the published results, it is therefore likely that MTs are important players in conferring heavy metal tolerance in *B*. *rapa*.

### Unique roles of MTs and PCs for heavy metal tolerance and homeostasis in plant

Although both MTs and PCs are known as heavy metal chelators, each MTs and PCs might have a distinct role in heavy metal homeostasis and detoxification. This might be particularly true for MTs, which are ubiquitously expressed in different organs of *B*. *rapa*. MTs might exhibit a prominent role for heavy metal homeostasis in plants grown on soils contaminated with low or trace levels of heavy metals. Under these conditions, PCs might exhibit a minor role for heavy metal homeostasis, due to the low activity of PC synthesis when heavy metals are present at low concentrations. Suspension cultured cells of *Rauwolfia serpentina* exhibit a non-detectable level of PCs in normal culture medium, but PC synthesis is highly induced when heavy metals are present at high concentrations, while GSH contents are gradually reduced proportionally to the increase of PCs synthesis [42]. Compared to other metals, Cd and Pb have the most pronounced effect for the induction of PC production [42], suggesting that specific PCs function for heavy metal detoxification under the heavy metal excess conditions and play only a minor role in heavy metal homeostasis.

In contrast to PCs, it is generally assumed that the main function of MTs is to contribute to metal ion homeostasis in plants growing under normal conditions. Interestingly, a dramatic increase of MTs transcripts was found in the phloem tissue of senescing leaves [43]. During the remobilization of heavy metals released from metalloproteins in senescing leaves into young tissues, MTs might stabilize the heavy metals to protect the plant from oxidative damage caused by the free form of a given heavy metal. Furthermore, they may contribute to the translocation of essential heavy metals during grain filling. Arabidopsis *MT4a* and *MT4b* are specially expressed in dry seeds [28], and barley metallothionein MT4 has been mainly detected in the embryo and aleurone layer of grains accumulating Zn [37]. MT4s harbors a strong Zn binding capacity and plays an important role in storing Zn in seeds to recycle Zn in germinated seedling. Actually, overexpression of Arabidopsis MT4a and MT4b enhances post germination growth, whereas knockdown of these genes caused the opposite result [28]. It is beyond any doubt that PCs are crucial in detoxifying heavy metals, but it needs to be discussed whether MTs are essential chelators to prevent damage caused by heavy metal toxicity. Arabidopsis *AtMT1a* and *AtMT2b* single or double knockout plants exhibit comparable growth as the WT under Cu and Cd excess conditions [45], while overexpression of *MT1* and *MT2* of *Brassica* species and pigeonpea increases Cd resistance in Arabidopsis [25–27]. The *mt1a-2 mt2b-1* mutant combined with *cad1-3* exhibits a higher sensitivity to Cu and Cd compared to the *cad1-3* mutant toxicity [44]. These results suggest that MTs are needed to confer metal tolerance and homeostasis in plants and that cooperation of MTs and PCs is necessary to protect plants efficiently from Cu and Cd toxicity.

Cd contamination in vegetables can poses a risk to human health. Brassica is a major leafy vegetable crop for human diet in many countries, therefore it might have a significant impact on the total Cd intake of humans [45]. Therefore, research aiming to reduce Cd accumulation in these vegetables is an important issue [46]. Although we know many aspects on heavy metal tolerance, we need a deeper understanding, since the role(s) of the different players appear to be complex as exemplified below for the *cad1-3* mutant. The Arabidopsis *PCS1* nonsense mutant *cad1-3* accumulate less Cd than the WT, while overexpression of *PCS1* genes induces Cd accumulation in yeast and plants [20, 47, 48]. Furthermore *cad1-3* transgenic Arabidopsis expressing either root-specific or ectopic expression of *TaPCS1* significantly enhance long-distance Cd transport into stems and rosette leaves, and reduce Cd accumulation in roots compared with *cad1-3* [47, 48]. These results indicate that PCS1s are useful gene resources to develop plants cleaning up environment contaminated with Cd, but not for developing vegetable crops with reduced Cd levels in their edible leafy parts. In contrast to chelators, Cd transporters located at vacuolar membrane in roots reduce Cd translocation from roots to shoots [46, 49]. Five full-length and four truncated haplotypes of the *BrHMA3*, *a gene* encoding a tonoplast-localized Cd transporter, affect the variation in the Cd root to shoot translocation among 64 accessions. Truncated *BrHMA3* haplotypes had 2.3- and 9.3-times higher shoot Cd concentrations and Cd translocation compared to the control [46]. Therefore, to develop leafy vegetable crops with low Cd and Cd tolerance, it needs to increase the capacity of both vacuolar compartmentation of PCs-Cd complex and Cd(II) ion in roots *via* molecular genetics and traditional breeding programs.

## Supporting information

S1 Table Primers used in this study. (DOCX)

S1 Fig Photography of gel showing inserts of cDNA library. (DOCX)

S2 Fig Alignment of CDS sequences of MT genes from Barssica species, *A*. *thaliana* and *T*. *caerulescens*. (DOCX)

S3 Fig Alignment of amino acid sequences of MT proteins from Barssica species, *A*. *thaliana* and *T*. *caerulescens*. (DOCX)

S4 Fig Alignment of amino acid sequences of PCS1 and PCS2 proteins from *Brassica* species, *A*. *thaliana*. (DOCX)

S5 Fig Phylogenetic analysis of MT genes. (DOCX)
