# Correction: Characterization of *Brassica rapa* metallothionein and phytochelatin synthase genes potentially involved in heavy metal detoxification

**DOI:** 10.1371/journal.pone.0263427

**Published:** 2022-01-27

**Authors:** Jiayou Liu, Jie Zhang, Sun Ha Kim, Hyun-Sook Lee, Enrico Martinoia, Won-Yong Song

The fifth author’s name is spelled incorrectly. The correct name is: Enrico Martinoia. The correct citation is: Liu J, Zhang J, Kim SH, Lee H-S, Martinoia E, Song W-Y (2021) Characterization of *Brassica rapa* metallothionein and phytochelatin synthase genes potentially involved in heavy metal detoxification. PLoS ONE 16(6): e0252899. https://doi.org/10.1371/journal.pone.0252899
