# Supplementary material for: Characterization of Brassica rapa metallothionein and phytochelatin synthase genes potentially involved in heavy metal detoxification
Source: PLoS One. 2021 Jun 4;16(6):e0252899. doi: 10.1371/journal.pone.0252899 (PMC8177407; doi:10.1371/journal.pone.0252899)
Supplement: S1 Table — (DOCX) [file pone.0252899.s001.docx]

S1 Table. primers used in this study.

| Primer | Sequence | Purpose | Ref. |
| --- | --- | --- | --- |
| CYC1 | AGGGCGTGAATGTAAGCGTGAC | pYES2 vector sequencing | This study |
| T7 | TAATACGACTCACTATAGGG | pYES2 vector sequencing | This study |
| BrMT1a-F | ACAAAGTACAACTTTAAATCAAAGAG | RT-PCR | This study |
| BrMT1a-R | CAATCACACAATCAACACATAGAAGC | RT-PCR | This study |
| BrMT1b-F | GCAAAAATTACAACTATTTTAAATC | RT-PCR | This study |
| BrMT1b-R | ACACCACACATATAAAGATATAAAGC | RT-PCR | This study |
| BrMT1c-F | CAAGTTTTAAACCAAAGAGAAGTAAG | RT-PCR | This study |
| BrMT1c-R | TACACAATAAACACCAAATACAGAG | RT-PCR | This study |
| BrMT2a-F | GAATAATGAAACCTTTCTAAGGAG | RT-PCR | This study |
| BrMT2a-R | GAATCATTCACGTTCATTCCATAG | RT-PCR | This study |
| BrMT2b-F | CAACCCCAATAAACCCAAACC | RT-PCR | This study |
| BrMT2b-R | TCATATATAGTCACACAGATAVAAC | RT-PCR | This study |
| BrMT3-F | GTCTTCGTGCGGAAACTGCGACTG | RT-PCR | This study |
| BrMT3-R | TATGAGCTCCATAGATGAACTCAC | RT-PCR | This study |
| BrPCS1-F | AACCTGCTTATTGTGGCTTGGC | RT-PCR | This study |
| BrPCS1-R | TATAGGTGAAAAGTGACCAGACC | RT-PCR | This study |
| BrPCS2-F | TGAGGATAAAGTGAAGGCTTACCC | RT-PCR | This study |
| BrPCS2-R | ATCAAATAGATCTCACAAGAGACC | RT-PCR | This study |
| BrActin-F | CATCAGGAAGGACTTGTACGG | RT-PCR | This study |
| BrActin-R | GATGGACCTGACTCGTCATAC | qRT-PCR | This study |
