# Supplementary material for: Characterization of Brassica rapa metallothionein and phytochelatin synthase genes potentially involved in heavy metal detoxification
Source: PLoS One. 2021 Jun 4;16(6):e0252899. doi: 10.1371/journal.pone.0252899 (PMC8177407; doi:10.1371/journal.pone.0252899)
Supplement: S1 Fig — (DOCX) [file pone.0252899.s002.docx]

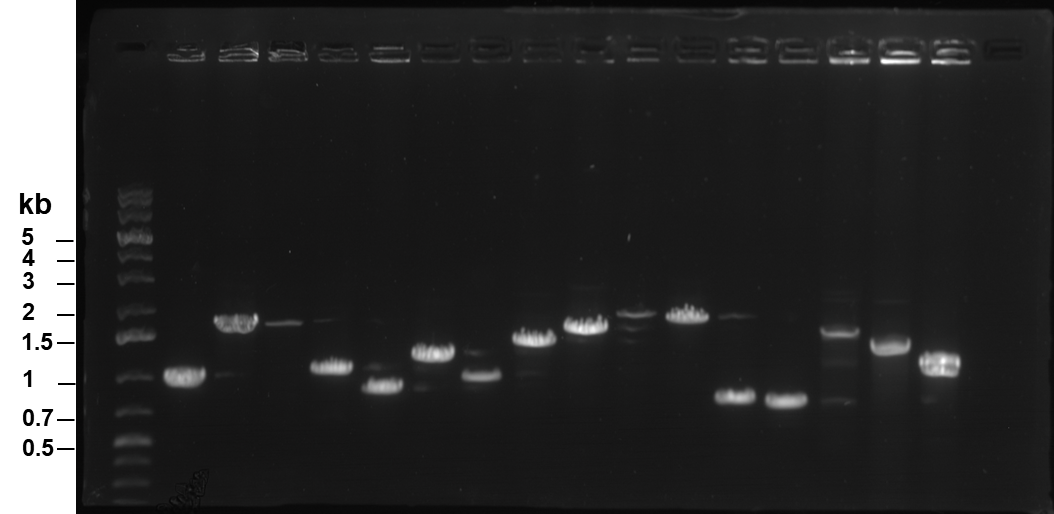


**S1 Fig. Photography of gel showing inserts of cDNA library.** Inserts of the *B, rapa* cDNA library in the pYES2 vector were amplified using PCR with vector linker primers, and the PCR products were electrophoresed in 1% agarose gel.
