# Supplementary material for: Characterization of Brassica rapa metallothionein and phytochelatin synthase genes potentially involved in heavy metal detoxification
Source: PLoS One. 2021 Jun 4;16(6):e0252899. doi: 10.1371/journal.pone.0252899 (PMC8177407; doi:10.1371/journal.pone.0252899)
Supplement: S2 Fig — (DOCX) [file pone.0252899.s003.docx]

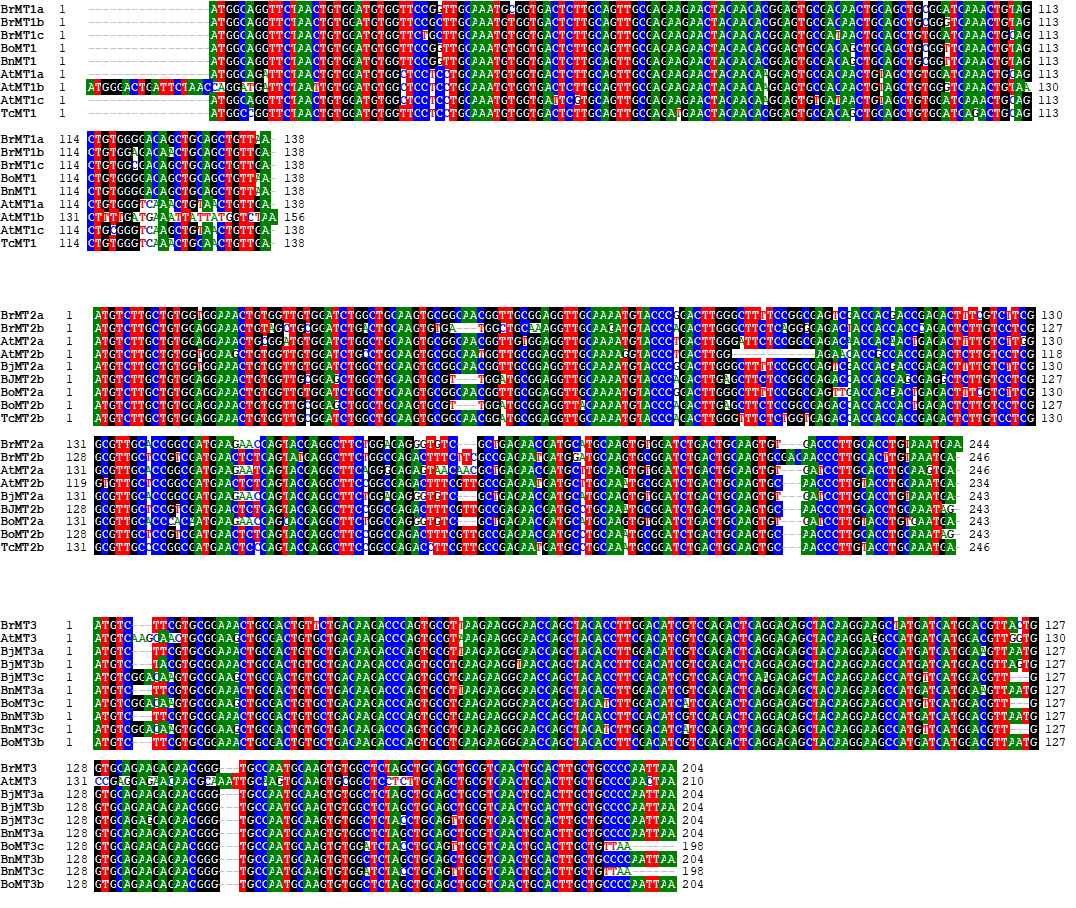


**S2 Fig. Alignment of CDS sequences of MT genes from Barssica species, A. thaliana and T. caerulescens.** CLUSTALW program of BioEdit was used for this alignment. Accession numbers of MTs are: S71334.1 for *BnMT1*, AF458412.1 for *BoMT1*, NM_100634.2 for *AtMT1c*, AF386921.1 for *AtMT1a*, NM_001037008.3 for *AtMT1b*, AY486004.1 for *TcMT1*, NM_111773.4 for *AtMT2a*, AK227568.1 for AtMT2b, Y10850.1 for BjMT2a, AF200712.1 for BoMT2a, XM_013767061.1 for *BoMT2-2b*, Y10851.1 for *BjMT2b*, AY486002.1 for*TcMT2b*, NM_112401.2 for *At MT3*, AB057413.1 for *BjMT3a*, AB057414.1 for *BjMT3b*, AB057415.1 for *BjMT3c*, XM_013847349.1 for *BnMT3a*, XM_013770994.1 for *BoMT3c*, XM_013842239.2 for *BnMT3b*, XM_013825697.2 for *BnMT3c*, XM_013782575.1 for *BoMT3b*
