# Supplementary material for: Characterization of Brassica rapa metallothionein and phytochelatin synthase genes potentially involved in heavy metal detoxification
Source: PLoS One. 2021 Jun 4;16(6):e0252899. doi: 10.1371/journal.pone.0252899 (PMC8177407; doi:10.1371/journal.pone.0252899)
Supplement: S3 Fig — (DOCX) [file pone.0252899.s004.docx]

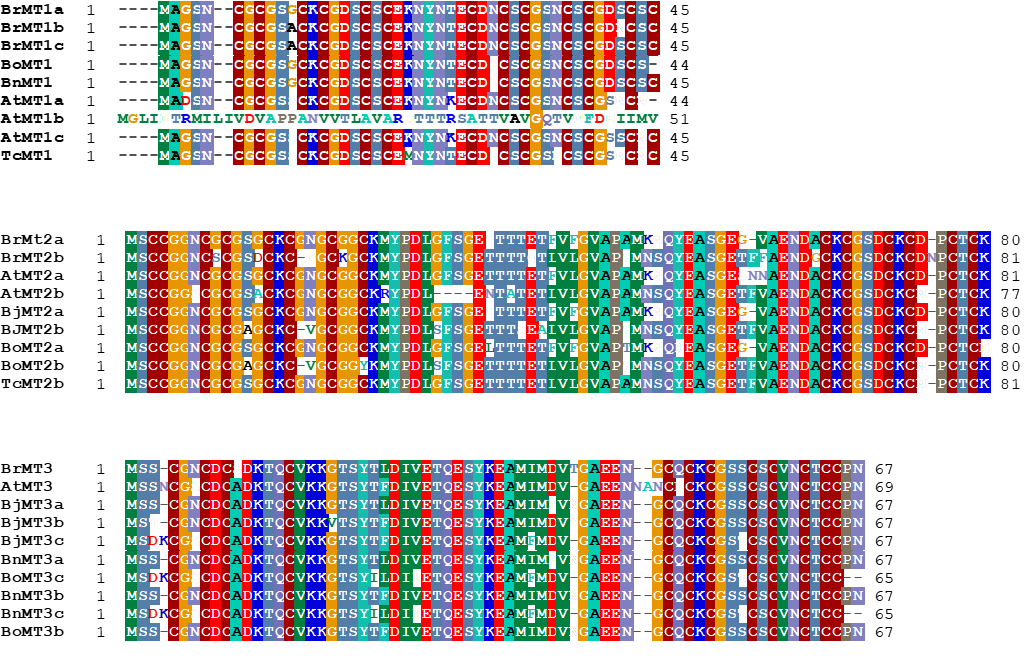


**S3 Fig. Alignment of amino acid sequences of MT proteins from Barssica species, A. thaliana and T. caerulescens.** CLUSTALW program of BioEdit was used for this alignment. Accession numbers of MTs are: MT361644 for BrMT1a, MT361645 for BrMT1b, MT361646 for BrMT1c, MT361647 for BrMT2a, MT361648 for BrMT2b, MT361649 for BrMT3, S71334.1 for BnMT1, AF458412.1 for BoMT1, NM_100634.2 for AtMT1c, AF386921.1 for AtMT1a, NM_001037008.3 for AtMT1b, AY486004.1 for TcMT1, NM_111773.4 for AtMT2a, AK227568.1 for AtMT2b, Y10850.1 for BjMT2a, AF200712.1 for BoMT2a, XM_013767061.1 for BoMT2b, Y10851.1 for BjMT2b, AY486002.1 forTcMT2b, NM_112401.2 for AtMT3, AB057413.1 for BjMT3a, AB057414.1 for BjMT3b, AB057415.1 for BjMT3c, XM_013847349.1 for BnMT3a, XM_013770994.1 for BoMT3c, XM_013842239.2 for BnMT3b, XM_013825697.2 for BnMT3c, XM_013782575.1 for BoMT3b
