# Supplementary material for: Characterization of Brassica rapa metallothionein and phytochelatin synthase genes potentially involved in heavy metal detoxification
Source: PLoS One. 2021 Jun 4;16(6):e0252899. doi: 10.1371/journal.pone.0252899 (PMC8177407; doi:10.1371/journal.pone.0252899)
Supplement: S4 Fig — (DOCX) [file pone.0252899.s005.docx]

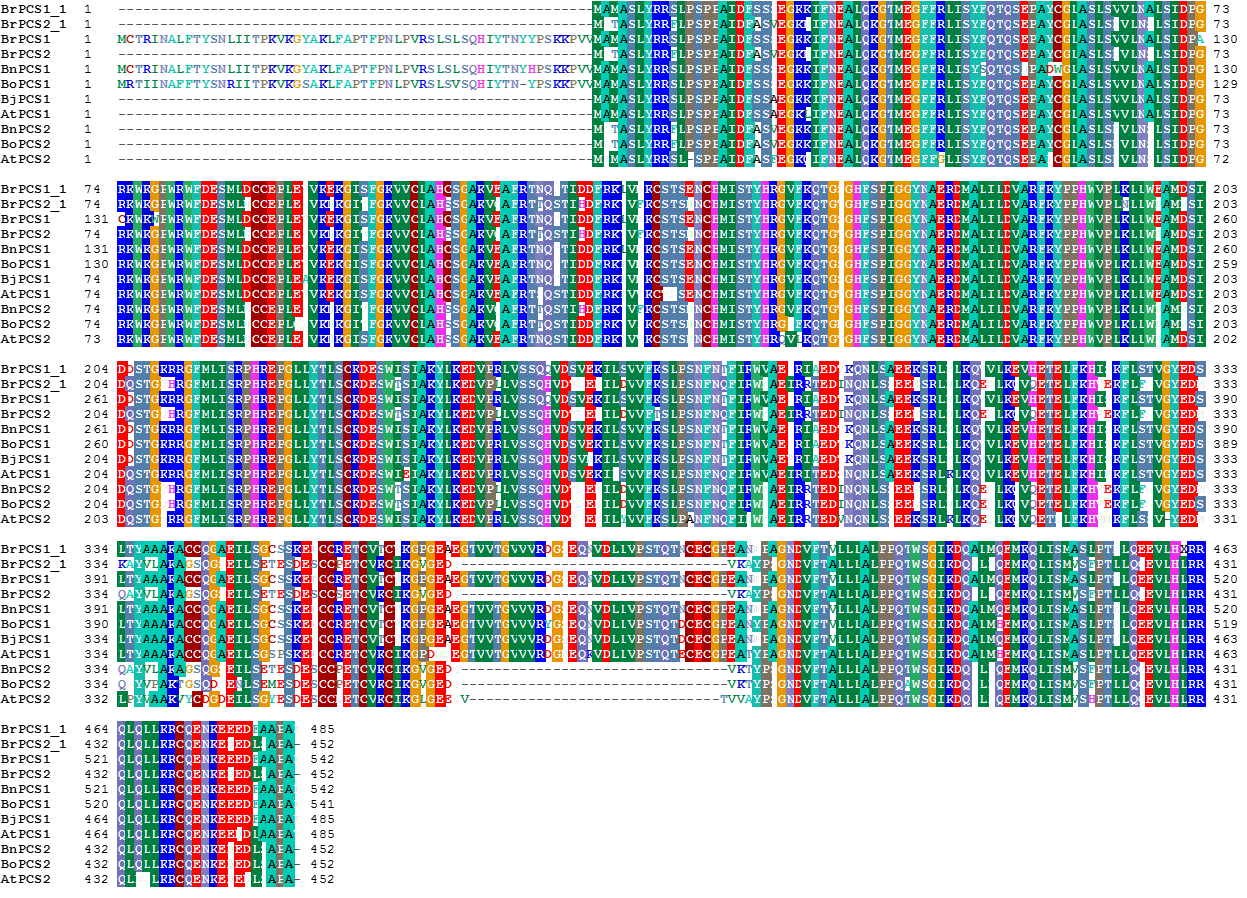


**S4 Fig. Alignment of amino acid sequences of PCS1 and PCS2 proteins from Brassica species, A. thaliana.** CLUSTALW program of BioEdit was used for this alignment. Accession numbers of PCS1 and PCS2: MT361642 for BrPCS1_1, MT361643 for BrPCS2_1, XM_009103491.2 for BrPCS1, XM_009120432.2 for BrPCS2, XM_013742676.1 for BnPCS1, XM_013742676.1 for BoPCS1, BAB85602.1 for BjPCS1, NP_199220.1 for AtPCS1, XP_013660298.1 for BnPCS2, XP_013602398.1 for BoPCS2, and NP_171894.1 for AtPCS2.
