# Supplementary material for: Characterization of Brassica rapa metallothionein and phytochelatin synthase genes potentially involved in heavy metal detoxification
Source: PLoS One. 2021 Jun 4;16(6):e0252899. doi: 10.1371/journal.pone.0252899 (PMC8177407; doi:10.1371/journal.pone.0252899)
Supplement: S5 Fig — (DOCX) [file pone.0252899.s006.docx]

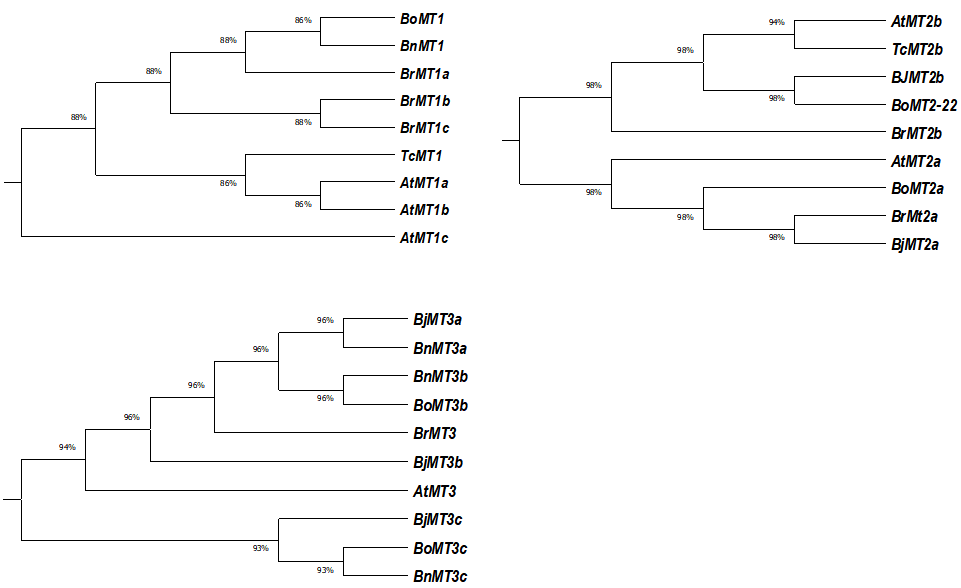


**S5 Figf. Phylogenetic analysis of MT genes.** Phylogenetic analysis using the neighbour-joining method implemented in MEGA X. The results are based on multiple alignments of MTs CDS DNA sequences. The bootstrap values (percentage) of 1000 replicates are shown at the branching points. Accession numbers of MTs are: S71334.1 for *BnMT1*, AF458412.1 for *BoMT1*, NM_100634.2 for *AtMT1c*, AF386921.1 for *AtMT1a*, NM_001037008.3 for *AtMT1b*, AY486004.1 for *TcMT1*, NM_111773.4 for *AtMT2a*, AK227568.1 for *AtMT2b*, Y10850.1 for *BjMT2a*, AF200712.1 for *BoMT2a*, XM_013767061.1 for *BoMT2b*, Y10851.1 for *BjMT2b*, AY486002.1 for *TcMT2b*, NM_112401.2 for *AtMT3*, AB057413.1 for *BjMT3a*, AB057414.1 for *BjMT3b*, AB057415.1 for *BjMT3c*, XM_013847349.1 for *BnMT3a*, XM_013770994.1 for *BoMT3c*, XM_013842239.2 for *BnMT3b*, XM_013825697.2 for *BnMT3c*, XM_013782575.1 for *BoMT3b*.
